# Quality of Diet Among Community-Dwelling Elders Participating in the Mobility and Vitality Lifestyle Program

**DOI:** 10.1093/geroni/igaa057.3081

**Published:** 2020-12-16

**Authors:** Jessica Cheng

**Affiliations:** University of Pittsburgh, Pittsburgh, Pennsylvania, United States

## Abstract

Dietary choices play an important role in disease prevention both through its effect on weight and independent of it. Improving diet can be an effective means of disease prevention among older adults. Participants (n=303) were recruited from the Allegheny County, PA area and received nutritional education in group sessions led by trained community health workers over one year. Diet quality was captured at baseline and final endpoint (either 9 or 13 months) using the Rate Your Plate (RYP) instrument for assessing healthfulness of diet and includes 24 items that can be summed to generate a total quality score. The mean RYP diet quality score improved from baseline (RYP=50.87) to endpoint (RYP=54.85) (p<.001). Over the course of the intervention, 30.9% of participants made enough improvement in diet to move to a better RYP category. A community-based group intervention for older adults was effective in inducing improvements in diet quality.

